# The Influence of Nutrients on Inflammatory Bowel Diseases

**DOI:** 10.1155/2020/2894169

**Published:** 2020-02-27

**Authors:** Sara Jarmakiewicz-Czaja, Dominika Piątek, Rafał Filip

**Affiliations:** ^1^Medical College of Rzeszow University, Rzeszow, Poland; ^2^Department of Conservative Dentistry with Endodontics, Medical University of Lublin, Lublin, Poland; ^3^Department of Gastroenterology with IBD Unit, Clinical Hospital No. 2, Rzeszów, Poland

## Abstract

Inflammatory bowel diseases is a group of inflammatory diseases. The pathogenesis of diseases is multifactorial, which may include a Western-type diet. Diseases occur with periods of recurrence and remission. Many factors can have a beneficial effect on reducing the frequency of recurrence and prolonging the remission period. Such ingredients include dietary fibre, mono- and polyunsaturated fatty acids, certain vitamins (D, C, and E), flavonoids, and minerals such as zinc and selenium. Properly selected nutrition might be an integral part of the treatment of patients with Crohn's disease or ulcerative colitis.

## 1. Introduction

The human digestive tract is responsible for various functions, including digestion, absorption, and assimilation of essential nutrients, but also for protection against pathogens. Its proper functioning is ensured by balance in the body. Deficiencies in certain components might lead to the development of various diseases. One of them might be inflammatory bowel disease (IBD). The main representatives of this group are ulcerative colitis (UC) and Crohn's disease (CD). It also includes other inflammatory diseases, for example, microscopic colitis.

IBDs are diseases that progress with periods of exacerbation and remission. During active disease, symptoms appear such as severe abdominal pain, diarrhoea with an admixture of blood and/or mucus, fever. In CD, inflammation can affect the entire length of the digestive tract. Most often, the disease process takes place in the final section of the small intestine and the initial section of the large intestine. The inflammation occurs alternately with healthy fragments. It affects the entire thickness of the gastrointestinal tract (mucous membrane, muscle fibres, and serosa), which is why fistulas are a common complication in this disease. In contrast, UC is characterized by an inflammatory condition that only affects the mucous membrane. The disease is located in the rectum and/or large intestine (most often the colon).

## 2. Pathogenesis

IBDs are diseases of unclear etiology. Numerous studies point to a multifactorial background, genetic, environmental, which include improper lifestyle, stress, and disorders of the immune system.

The genetic basis is one of the most important causes of the development of inflammatory bowel diseases. Numerous studies show that patients with CD are more likely to have a family history of this disease than those with UC. It should be emphasized that both disease entities are subject to the probability of occurrence in the family. Mutations occur mainly in genes that are responsible for the body's normal immune response. Genes that are responsible for identifying repetitive molecular patterns of pathogens can be distinguished. They may be related to the construction or metabolism of pathogenic microorganisms. One can distinguish among others bacterial lipopolysaccharides, peptidoglycans, or bacterial wall proteins. Examples of such genes include NOD2, TLR, and OCTN1/2. TLR is associated with an increased risk of both disease entities, while the others mainly with CD [1]. Another group of genes are associated with autophagocytosis, whose main function is to maintain appropriate intestinal epithelium homeostasis and regulate the inflammation of the body. The main autophagy genes important in inflammatory bowel diseases are NOD2, ATG16L1, and IRGM [2]. Mutations of the IL23R, IL12B, and STAT3 genes may increase the risk of IBD [3].

An abnormal reaction of the immune system to commensal bacteria causes a change in the intestinal microflora. The intestinal microflora is different for each person and depends primarily on lifestyle. However, even when exposed to various adverse environmental factors, the body tries to maintain balance. As a result of improper changes, intestinal dysbiosis may occur, which causes the body to reduce the beneficial commensal bacterial in favour of microorganisms that are considered to adversely affect the human body. This may have an impact on the development of IBD [4]. Changes have been demonstrated in the intestinal microflora between patients with IBD and control group. In healthy individuals, bacteria such as *Firmicutes* and *Bacteroidetes* dominate. Differences are also observed between periods of relapse and remission of the diseases as well as between the disease entities themselves. In patients with CD, more numerous *Streptococcus* spp. bacteria were observed, while in UC it was *Lactobacillus* spp. However, in the period of exacerbation in CD, *Bacteroidetes* and *Fusobacteria* were more numerous, in contrast to UC, where in the inflammation *Proteobacteria* and *Firmicutes* were more often observed [5]. An additional factor that may predispose to IBD is the Western-type diet. Many studies have shown this type of nutrition, which contains a greater amount of processed foods, including refined carbohydrates and saturated fatty acids, as acting adversely on the intestinal microflora, which can cause enteritis [6].

However, patients can benefit from using the Mediterranean diet due to its ingredients that have anti-inflammatory and reduction of intestinal permeability effects [7].

## 3. Nutritional Influencing Factors in Inflammatory Bowel Diseases

### 3.1. Dietary Fibre

According to the definition of the American Association of Cereal Chemists (AACC), dietary fibre is “the edible parts of plants or analogous carbohydrates that are resistant to digestion and absorption in the human small intestine with complete or partial fermentation in the large intestine” [8]. Depending on the composition, we distinguish two fractions of digestive fibre: soluble and insoluble in water.  Table 1 presents the composition and functions of particular fibre fractions.

According to numerous studies, dietary fibre is important in the pathogenesis of IBD. The products that are produced by microbial fermentation of dietary fibre are mainly acetate, propionate, and butyrate. It has been shown that intestinal bacteria producing short-chain fatty acids (SCFA), as products of their metabolism, can reduce inflammation by, among others, lowering the concentration of proinflammatory cytokines [10]. Bacteria that produce SCFA are beneficial to the body as they inhibit the growth of pathogenic bacteria, e.g., *Enterobacteriaceae* [11]. Numerous studies indicate the beneficial effects of dietary fibre in maintaining the proper function of the intestinal barrier, which protects the body against the entry of pathogens [12, 13].

During exacerbation of the disease, a diet with a restriction of the insoluble fraction is recommended in order to protect the disease-affected gastrointestinal tract from mechanical irritation. Ananthakrishnan et al. indicated a protective effect of dietary fibre of the soluble fraction on the maintaining of the intestinal barrier at an appropriate level. The authors describe too the reduction of the risk of CD after eating fibre from fruit, while cereals and dry legume seeds did not alter this risk. However, they did not show such a connection with the risk of developing UC [14]. Andersen et al. did not observe a connection between dietary fibre of the soluble and insoluble fractions with the risk of developing IBD [15]. In the latest Cochrane review, the authors point out that there is insufficient evidence that disease activity is associated with varied amounts and fraction of dietary fibre [16].

Further research is needed on the influence of dietary fibre of various fractions on the risk of occurrence and the course of IBD.

### 3.2. The Influence of Fats on the Development and Course of Inflammatory Bowel Diseases

Fats are a source of energy, a carrier of flavour, body-building components, and carriers of fat-soluble vitamins (A, D, E, and K); they inhibit gastric contractions and emptying. Fat is the main source of energy storage in the form of adipose tissue (triglycerides). They can have both beneficial and adverse effects on the body due to the composition and proportions of fatty acids taken with food.

Fatty acids are divided according to their structure into saturated fatty acids, trans-fatty acids, monounsaturated fatty acids, and essential unsaturated fatty acids. Products that are good sources of fatty acids are shown in Table 2.

Trans-fatty acids that have been produced by industrial methods increase the risk of cardiovascular disease. They reduce the flexibility of cell membranes, which adversely affects cell function and may predispose toward atherosclerosis. It has also been proven that the consumption of large amounts of trans-fatty acids increases the risk of certain cancers, including colon and breast [18, 19]. The main sources of these acids in human diets are highly processed products such as margarines, crackers, pretzel sticks, crisps, and biscuits. According to the nutrition standards for the Polish population from 2017, trans-fatty acids should be in an amount “as low as it is possible to achieve in a diet providing the right nutritional value” [20]. Jazernik et al. indicate the importance of these fatty acids in the pathogenesis of inflammatory bowel disease [21].

Not only are saturated fatty acids building material for tissues, but they are also the main form of energy reserve for the body. They can increase LDL (low-density lipoprotein) in the blood, which also predisposes toward the development of cardiovascular disease. Like trans-fatty acids, they increase the risk of cancer [19]. Numerous studies show that in the case of IBDs, the intake of a large amount of saturated fatty acids may increase the risk of their occurrence [22]. Jazayeri et al. described in their work the importance of various factors on the composition of the intestinal microflora. Bacteria that are predominantly found in healthy people, e.g., *Bacteroidetes* and *Firmicutes*, are present in people who follow a diet that is low in fat and high in dietary fibre. The intestinal microflora may be disadvantageous when fats (SFA) are dominant in the diet [23]. However, current scientific reports indicate uncertain connections between products such as red meat and its derivatives, which are a significant source of SFA, and disease activity. Further research in this direction is needed [16].

The mono- and polyunsaturated fatty acids are antiatherosclerotic, and they are also building blocks of the body. Due to their properties, they are able to act as anti-inflammatories. They participate in the proper functioning of the circulatory, nervous, and digestive systems. They must be delivered to the human body in food [24]. Lee et al. described the importance of polyunsaturated fatty acids for the risk of developing tumours. Omega-6 (o-6) has proinflammatory effects, while omega-3 (o-3) has anti-inflammatory effects. The amounts of fatty acids taken in and the ratio of o-6 to o-3 are important [25]. A large study from 2013 conducted by Ananthakrishnan et al. describes the protective effect of o-3 on the occurrence of UC [26]. The anti-inflammatory effects of acids belonging to the o-3 family, docosahexaenoic acid (DHA) and eicosapentaenoic acid (EPA), are mainly related to the reduction of prostaglandin production from arachidonic acid, proinflammatory cytokines, and high activity of T lymphocytes. Calder indicates that anti-inflammatory effects can be seen only after introducing a minimum of 2g of EPA and DHA per day into the diet [27]. Numerous studies prove that o-3 fatty acids and their appropriate ratio to o-6 can favourably reduce the inflammatory markers in the large intestine. Therefore, many studies also indicate the beneficial effect of o-3 above all in UC [28, 29]. However, fats (in total) consumed in excess may increase the risk of both CD and UC [30].

### 3.3. Vitamins

#### 3.3.1. Vitamin D

Vitamin D is one of the four fat-soluble vitamins. The hormonal form of vitamin D-1,25-dihydroxy vitamin D3 (1.25D) is particularly important for the immune system. Certain cells in the immune system have been shown to have nuclear receptors for 1.25D (VDR). Numerous studies indicate a connection with T, B lymphocytes, monocytes, and macrophages. Wang et al. have shown that the active form of vitamin D can induce the gene expression of cathelicidin antimicrobial protein (CAMP) in monocytes and *β*-defensin 2 (DEFB 4) [31]. Reduction of serum vitamin D concentration may increase the risk of infection [32–34]. Dionne et al. studied the effect of vitamin D in CD patients. They showed that 1.25D significantly reduced the proinflammatory activity of M1-type macrophages but did not contribute to a reduction of the anti-inflammatory effect of M2 macrophages. The level of secreted anti-inflammatory IL-10 was not affected [35]. Vitamin D also significantly affects the NOD2 gene, the mutation of which is associated with CD. Wang et al. indicate a strong association of 1.25D with the induction of NOD2 expression, which in turn suggests that vitamin D deficiency may also contribute to the occurrence of this disease [32]. Researchers often indicate the co-occurrence of vitamin D deficiencies in patients with IBD. In one study conducted by Ananthakrishnan et al., it was shown that one-third of IBD patients have a deficiency in this vitamin. They showed that its low level correlated with the incidence of colon cancer in patients with IBD [36]. Deficiencies in this group are most often caused by too low intensity of sunlight and the duration of the active phase of the disease. It was found that the amount of one of the forms of vitamin D-25(OH)D3 in the serum at 30 ng/ml can inhibit the secretion of proinflammatory cytokines (IL-6 and TNF-*α*) induced by lipopolysaccharide (LPS), which is a component of bacterial walls [37, 38]. Due to the low costs of supplementation and the immunostimulatory properties of the component, the vitamin should be introduced into the diet in a pharmacological form. Proper diet is also important. Products that are good sources of vitamin D are shown in Table 3.

#### 3.3.2. Vitamin C

Vitamin C is a water-soluble compound. It occurs in two forms: L-ascorbic acid and the product of its oxidation, L-dehydroascorbic acid. It is a powerful antioxidant; hence, it protects against reactive oxygen species. Vitamin C shows increased concentration in neutrophils, exceeding the plasma concentration, which protects them against the action of free radicals. It also increases chemotaxis and chemokinesis of these cells [40]. Schwager et al. showed that L-ascorbic acid can improve the function of leukocytes [41]. The researchers point to the accumulation of ascorbic acid in lymphocytes and monocytes [42]. Elste et al. recommend saturation of the plasma with ascorbic acid ≥70 *μ*mol/l to obtain neutrophil saturation with the compound and ensure proper functioning of the immune system [40]. Vitamin C is also present in significant amounts in lymphocytes and in phagocytes; in addition, it also increases the proliferation of T lymphocytes. Given the above information, it seems necessary to use ascorbic acid in IBD. A properly balanced diet can contribute to an increase in the body's resistance. Berries, citrus fruits, potatoes, parsley, and cruciferous vegetables are rich dietary sources of vitamin C. IBD patients are advised against the latter due to the severe effects on the body of their difficulty in digestion, especially during exacerbation of the disease.

#### 3.3.3. Vitamin E

Vitamin E belongs to the group of fat-soluble vitamins. It includes eight different compounds: four tocopherols and four tocotrienols. It is a powerful antioxidant of, among others, polyunsaturated fatty acids. It prevents degenerative diseases that are mainly associated with the aging process. It has a beneficial effect on the circulatory system, and it can also prevent tumours [43]. Patients with IBD, as a result of the active phase of the disease, treatment or limitation of food intake, may show many deficiencies. A meta-analysis presented by Fabisiak et al. shows numerous deficiencies of fat-soluble vitamins, including vitamin E [44]. Oxidative stress is associated with inflammatory bowel diseases, which increases the need for antioxidants in these patients, especially during exacerbation of the disease [45, 46]. Chen et al. also indicate that vitamin E has a beneficial effect on the inhibition of colorectal cancer in mice by reducing the proinflammatory mediators in tumour cells and neighbouring cells [47]. This component protects the DNA against damage [48]. In addition, it has anti-inflammatory and antioedema effects on the skin, which may be particularly beneficial in the case of extraintestinal symptoms of inflammatory bowel diseases [49]. Vitamin E is found mainly in vegetable oils, e.g., rapeseed oil (26.73 mg/100 g), olive oil (11.95 mg/100 g), and butter (2.52 mg/100 g) [40].

#### 3.3.4. Vitamin B2 (Riboflavin)

It is an organic compound consisting of isoalloxazine and ribose. It is absorbed in the upper section of the small intestine, where it is transformed into flavin coenzymes. They occur in two biologically active forms: flavin mononucleotide (FMN) and flavin adenine dinucleotide (FAD).

Riboflavin is involved, among others, in the process of glucose oxidation, and in the degradation and synthesis of bile acids. It is important in the transformation of retinol to retinoic acid. It also participates in the proper functioning of the circulatory, nervous, and immune systems. Vitamin B2 can inhibit the release of TNF-*α* as well as support the normal response of macrophages.

In their study, Anandam et al. showed that as a result of chronic inflammation and bacterial infection in the intestinal mucosa, the level of proinflammatory cytokines increases, and thus the absorption of vitamin B2 from food is reduced. Decreased uptake is associated with prolonged exposure of the intestine to TNF-*α* [50]. Riboflavin is a compound that can be synthesized by the intestinal bacterial microflora. De Moreno de LeBlanc et al. in their work describe the importance of supplementation of lactic acid bacteria, which may support the process of treating IBD patients by producing certain vitamins, including vitamin B2, which has a positive effect on the immune system [51].

Rich sources of riboflavin in food are baker's yeast, milk and dairy products, meat, and eggs. Inadequate storage (UV rays, acidic and alkaline environments) can reduce its amount in food products by up to 85%.

#### 3.3.5. Vitamin B6 (Pyridoxine)

Pyridoxine belongs to the group of water-soluble vitamins. It occurs in three forms: pyridoxal, pyridoxamine, and pyridoxine. Each form can be phosphorylated, but pyridoxal-5-phosphate (PLP) is the most biologically active compound [52].

It is a coenzyme found in 60 enzymes. It plays an important role in the metabolism of amino acids. It also participates in the conversion of fats and carbohydrates. It is necessary for the synthesis of serotonin and niacin from tryptophan. It supports the proper functioning of the immune system. It is also synthesized by the intestinal microflora.

Food products that are a good source of pyridoxine are presented as follows:

- Yeast
- Wheat bran
- Legume seeds
- Meat
- Milk
- Eggs

Many food treatment processes can cause a reduction of pyridoxine in food products. The biggest losses are observed during heat treatment, grain milling, and freezing.

Decreased blood levels may be associated with the occurrence of inflammation in the body. Selhub et al. showed in their research that supplementation with vitamin B6 can reduce inflammation. The research was conducted on mice, and the authors indicate the need to continue research in this area [53]. IBD patients have also demonstrated a decreased level of pyridoxine compared to a control group, which may be useful information for planning nutritional strategies [54].

#### 3.3.6. Vitamin A

Vitamin A is a fat-soluble compound. We distinguish various forms of this vitamin, among others: retinol, retinal, retinoic acid (RA), and carotenoids. The immunoactive form of vitamin A—retinoic acid—may have an effect on increasing T-cell proliferation [55]. Yin et al. discuss improving the CD8 T-cell immune response [56]. Retinoic acid may also be associated with increased IL-10 secretion [57]. RA is also associated with the synthesis of TGF-*β* (transforming growth factor *β*), which has anti-inflammatory properties [58]. However, the action of RA depends on its concentration in the body. Grizotte-Lake et al. showed in their study that bacteria of the genus *Clostridia* can differentiate RA concentration by modulating the expression of Rdh7 (retinol dehydrogenase 7) [59]. RA also has effects on DC (dendritic cells) and gastrointestinal mucosa CD103 receptors as well as CX3CR1, which, among others, regulates intestinal macrophage homeostasis [60]. Retinol has anti-inflammatory effects by reducing IFN*γ* and IL1*β* in fibroblasts [61]. Retinoids also take part in the synthesis of secretory epithelium, whereas they have an inhibitory effect on the formation of keratinized epithelium [62, 63].

#### 3.3.7. *β*-Carotene

*β*-Carotene belongs to a group of 700 carotenoids. 60 of them are found in food, while 20 can be identified in the blood [64]. They are substances that are insoluble in water but are fat-soluble. *β*-Carotene is one of the most biologically active ingredients with an antioxidant effect on the human body. The human body cannot synthesize its compounds alone, unlike plants, and therefore it is necessary that it is supplied with food. It is oxidized in the small intestine by *β*-carotene dioxygenase and then transferred via lipoproteins to the tissues. *β*-Carotene accumulates mainly in adipose tissue. This component shows the highest biological activity among the whole group of carotenoids, because its structure is similar to retinol (it is made of two *β*-ionone rings). Products that are good sources of *β*-carotene in vegetables are shown in Table 4 and in fruits Table 5.

Under the influence of heat treatment, the bioavailability of *β*-carotene increases, while its antioxidant activity is not reduced. The amount of absorbed ingredient depends on the type of food being taken and the digestive capacity of the body [65].

The bioavailability of an antioxidant is also influenced by factors that increase or decrease its absorption from food. Fat is one of the ingredients that aid its absorption, but dietary fibre and phytosterols, among other things, have a limiting effect.

*β*-Carotene has a strong antioxidant effect by capturing oxygen and binding ions through which free radicals are formed [64]. It can also interact with tocopherol and vitamin C, enhancing their function, which can delay the body's aging processes [65]. Numerous studies prove that it also has anticancer properties [66–68]. The authors emphasize its role in supporting the proper functioning of the immune system, e.g., after exposure to UV radiation. Increased NK cell activity was also shown after supplementation with *β*-carotene. The intensity of the immune response depends on the number of MHC II positive monocytes [69].

Fabisiak et al. showed in their meta-analysis significant deficiencies of vitamin A in CD and in UC as compared to a control group. It is important that patients, especially with the active form of the disease, should have an adequate supply of fat-soluble vitamins, including *β*-carotene [46]. D'Odorico et al. conducted a study in which, among others, levels of carotenoids and tocopherol were measured in patients with IBD and in a control group. They showed a reduced concentration of antioxidants in patients with CD and UC, regardless of disease activity. A particularly low level was found in malnourished patients. The results obtained may have been due to the use of an inadequate diet by the patients or the active form of inflammatory disease [70]. Geerling et al. came to similar conclusions when carrying out a comprehensive study of nutritional status in patients newly diagnosed with CD and UC. The concentration of components such as *β*-carotene, zinc, or selenium was lower in patients with UC compared to a control group. These patients also showed other deficiencies of vitamins (cobalamin and riboflavin) or minerals (calcium, magnesium, and phosphorus) necessary for the proper functioning of the body [71]. It is necessary to perform diagnostics for nutritional deficiencies in newly diagnosed patients and those with long-term illness. Kaulmann et al. in their work described the relationship between carotenoids and the occurrence of chronic inflammatory diseases. They described the modulation of inflammatory stress pathways. An example is the blocking by carotenoids of the translocation of the nuclear factor (NF-*κ*B) to the nucleus, which in turn causes the production of proinflammatory cytokines to be inhibited [72]. Hong et al. came to similar conclusions, describing the alleviation of inflammation of the large intestine by inhibiting NF-*κ*B signalling after administration of a compound from the group of carotenoids (all-trans retinoic acid) [73]. The above studies prove that it is important that the diets of patients with IBD include ingredients that are a good source of carotenoids. However, it should be remembered that when the disease is exacerbated, with co-occurring diarrhoea, the supply of fats should be reduced; thus, the bioavailability of carotenoids will be reduced. Therefore, it is important to ensure a sufficient amount and proper choice of food products that are sources of carotenoids (they should be vegetables or fruits that are easily digestible).

### 3.4. Minerals

#### 3.4.1. Zinc

Zinc is a food component classed as a micronutrient. This element is necessary for the proper functioning of certain enzymes and hormones. It is an essential component for the synthesis of proteins and erythrocytes. It is also responsible for the proper functioning of the immune system. Its absorption takes place in the small intestine. Absorption of the component from food products only takes place up to 40%. In food, many nutrients have an effect on the uptake of zinc. The absorption of zinc is increased by the presence of an appropriate amount of complete protein in the diet. In contrast, polyphosphates, phytates, or excessive consumption of such elements as iron or calcium may reduce the absorption of this micronutrient [74, 75]. An insufficient supply of zinc in the diet has been shown to cause a number of abnormalities in the body. It can lead to stunted growth and a reduced rate of development. Deficiency also correlates with depressed mood and depression, especially in people with IBD [76]. Deficits may also cause skin lesions or worsened wound healing.

The effect of zinc on the immune system is significant. A reduced concentration of the element contributes to reduced monocyte adhesion activity. In addition, its deficiency may increase the secretion of proinflammatory cytokines, mainly IL-6. This state in the body also contributes to atrophy of the thymus as well as reducing the number of mature T lymphocytes [77]. An appropriate level of zinc is important for the activation of T cells and their proper functioning [78]. In animal studies, zinc deficiency has been shown to reduce the production of IL-2, a factor that influences the growth of mainly cytotoxic T lymphocytes [78]. A deficiency may cause apoptosis of mature B lymphocytes and reduce the number of immature B lymphocytes. This leads to a reduction in the production of immunoglobulins, which has a significant effect on the lowering of immune system functioning [79]. Its concentration also affects NK cells that belong to the lymphocyte family. IL-12 is a factor that increases the effect of NK cells. It has been observed that decreased zinc concentration reduces NK activity. Low values of the element in the body may lead to the disruption of the phagocytic capacity of macrophages [80].

In patients with IBD, zinc deficiency may be caused by chronic diarrhoea and intestinal inflammation, as a result of which the element cannot be absorbed in sufficient quantity. Reduced amounts have been shown in the plasma of patients. Ananthakrishnan et al. showed that higher intake of zinc was associated with a reduction in the risk of CD but had no effect on the occurrence of UC. A proper amount of zinc may also contribute to the prolongation of a remission period of the disease [81].

#### 3.4.2. Selenium

Selenium is one of the essential minerals that must be delivered into the body with food. In the body, it occurs mainly in the skeleton, kidneys, and liver. The bioavailability of the micronutrient from food products is up to 80% and absorption takes place mainly in the duodenum, but also in the colon. Selenium has an antioxidant effect and protects cells against the adverse effects of free radicals. It is involved in the conversion of thyroid hormones, regulates growth processes, and is a component of many enzymes necessary for the proper functioning of the body (e.g., glutathione peroxidase and selenophosphate synthetase). A deficiency of this element can lead to muscle damage, dystrophy of articular cartilage, or reduction of the body's immunity through, among others, B-lymphocyte dysfunction or decreased NK cell activity [82]. However, an excess of selenium supply may lead to selenosis, which is manifested by depression, vomiting, or inflammatory skin conditions. IBD is characterized by ongoing chronic inflammation, which generates, among others, oxidative stress. Selenium deficiency has been demonstrated during both exacerbation and remission in patients with UC and CD [83]. Researchers describe selenium deficiencies in patients with CD, which may lead to the reduction of the body's immune response to the development of inflammation. Han et al. indicate female sex, corticosteroid use, and inflammation as the main factors that may predispose toward deficiencies of this element [84]. Similar conclusions related to the occurrence of deficiencies in women have been shown by Aguilar–Tablada et al. They also presented results that show a correlation between low selenium level and BMI of patients (a lower level was demonstrated in patients with BMI <18.5) [85].

The main sources of selenium in the human diet are cereal products, sprouts, meat, milk and its products, and fish [86].

#### 3.4.3. Iron

**Figure 1:**
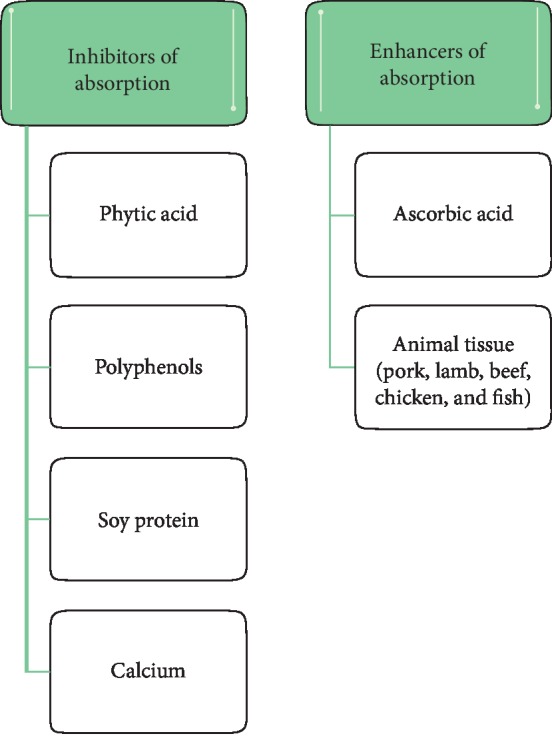
Factors that increase and reduce the absorption of iron [89].

This is a compound that is one of the microelements. In the human body, it is found in myoglobin, haemoglobin, and transferrin and is a component of many enzymes. In the stomach, due to the acidic environment, iron is partially ionized from trivalent to divalent. In an alkaline environment, e.g., when using antacids, its bioavailability is limited. The ingredient is absorbed in the small intestine and stored in the liver, kidneys, blood serum, spleen, and bone marrow [87]. The bioavailability of heme iron in the small intestine is up to 20%, while nonheme iron is up to 5% [88]. It is correlated with the composition of the diet. Some food components show properties reducing or increasing its absorption (Figure 1). Physical health, medications, and dietary supplements also have an impact.

Below, food products that are a good source of iron are presented, divided into two fractions.

Iron heme sources in the diet:

- Meat
- Fish
- Poultry
- Offal (liver, kidney, and heart)

Nonheme iron sources in the diet:

- Whole-grain cereal products
- Green vegetables (spinach, parsley)
- Dried fruits
- Dried vegetables

It transports oxygen and is involved in the metabolism of cholesterol, tyrosine iodination, and synthesis of DNA (deoxyribonucleic acid). It also has a significant impact on the proper functioning of the immune system. A deficiency of the element may lead to microcytic anaemia. It can manifest itself in paleness of skin layers, lack of concentration, rapid fatigue, and reduced immunity of the body.

Anaemia is one of the most common complications in IBD, both from iron deficiency and due to chronic diseases. It is often associated with a decrease in quality of life in these patients due to the symptoms that appear. The causes for these patients may include gastrointestinal bleeding, reduced iron absorption, resection of the intestine, and the exclusion of some food groups by patients [90]. Mucke et al. in their literature review indicate the necessity of screening for anaemia in patients with active disease at least once every three months, while patients in remission are recommended to be examined every 6–12 months [90]. A study on the incidence of anaemia in IBD patients published by ECCO (European Crohn's and Colitis Organisation) showed that in 42% of them anaemia was found in the first year after diagnosis. It is more often found in CD patients than in UC patients [91]. The authors of numerous studies describe the relationship of hepcidin-to-iron concentration [92]. Hepcidin is a compound produced in the liver which participates, inter alia, in the regulation of iron homeostasis in the body. It combines with ferroportin, which reduces the absorption of iron. It also significantly reduces the availability of iron stored in the liver and macrophages [93]. In many studies, increased hepcidin levels have been shown in IBD patients [94, 95]. Moran-Lev et al. presented a reduction in hepcidin concentration after vitamin D supplementation in children diagnosed with inflammatory bowel disease. This, in turn, may be an important factor supporting the treatment of anaemia among these patients [96]. In addition, Castro et al. describe in their work a possible relationship between intestinal dysbiosis (increasing the amount of *Proteobacteria* and reducing *Firmicutes* and *Bacteroidetes*) and a reduction in the effectiveness of anaemia treatment by oral iron supplementation [97].

#### 3.4.4. Magnesium

It is an element whose content in the human body is 22–26 g. More than half of the content is in the skeletal system, 40% in muscles and soft tissues, and 1% in red blood cells and plasma [98].

It is a catalyst for the conversion of proteins, fats, and carbohydrates. It is one of the building components of the body. It takes part in regulating blood pressure and nerve conduction. It also has a significant influence on the synthesis of high-energy bonds (ATP and GTP). In addition, it ensures the maintenance of the integrity of biological membranes such as mitochondria and lysosomes and is necessary for the active transport of certain minerals.

The daily requirement for this element is 300–400 mg/day for adults. However, the normal concentration of magnesium in the blood should be from 0.65 to 1.25 mmol/l [99].  Table 6 presents the good sources of magnesium.

Absorption of magnesium from food products can be reduced by the high content of minerals such as calcium, phosphorus, and zinc in the diet. In addition, phytate and oxalic acid may impair its bioavailability. Karmańska et al. in their literature review indicate proteins as a factor increasing the absorption of the element from food [98].

In IBD, many authors indicate a strong association between serum magnesium and inflammation. Trapani et al. in their study evaluated the role of magnesium in modulating dextran sulphate sodium (DSS)-induced colitis in mice. After the introduction of magnesium supplementation, a reduction in inflammation was obtained by decreasing the effect of TNF-*α* [100]. Naser et al. also indicate the possible role of hypomagnesaemia in the pathogenesis of IBD [101]. Taylor et al. in their study aimed at assessing the intake of selected food components; they assessed that patients with IBD showed a lower intake of magnesium compared to a control group [102]. Szczuko et al. also suggest an insufficient supply of magnesium in the diet of IBD patients [103].

When assessing the diets of people with IBD in terms of magnesium content, one should remember about the appropriate selection of food products. Not all products that are a rich source of this element can be recommended to the patient during a period of exacerbation of the disease. In the case of large deficiencies, supplementation should be considered.

### 3.5. Flavonoids

Flavonoids are compounds that belong to a large group of polyphenols. They are divided according to their construction into

- Flavanols
- Flavones
- Flavanones
- Isoflavones
- Flavonols
- Anthocyanins [104]

Flavonoids have an antioxidant action and they limit the production of free radicals in cells, inhibit lipid oxidation, or bind transition metal ions. They may limit the synthesis of proinflammatory factors (proinflammatory cytokines). An antiallergic effect has also been demonstrated due to their inhibition of the secretion of histamine from mastocytes—mast cells that are activated for secretion by prostaglandins and cytokines. Flavonoids inhibit, among others, myeloperoxidase (MPO), which indirectly affects the cytotoxicity of intestinal tissues. It has been shown that giving, among others, quercetin and rutin has beneficial effects for IBD patients. The best sources of quercetin and rutin are chokeberry, elderberry fruit, and capers. Vezza et al. describe remission in patients with IBD after giving flavonoids, but only in experimental models. It is necessary to continue research in this area [105]. Oteiza et al. describe the beneficial effects of flavonoids on the intestinal microbiota. They may inhibit pathogenic microorganisms such as *Bacillus cereus, Campylobacter jejuni,* and *Lactobacillus* sp. In addition, they can increase the microbiota beneficial to the host in the form of *Bifidobacterium* spp. The effect of flavonoids on the intestinal microflora depends on the type and amount of the given component [106]. Flavonoids are found in many vegetables (broccoli, celery, root parsley, and tomatoes), fruits (apples, red grapes, oranges, strawberries, blueberry, and chokeberry), green tea, and olive oil.

### 3.6. Bioactive Compounds

#### 3.6.1. Lactoferrin (LF)

This is a glycoprotein belonging to the group of transferrins. It shows iron-binding properties. It occurs in secretions of the human body; significant amounts are observed in the colostrum of human milk (9 g/litre). However, its antibacterial values are less than that of other mammals' milk [107]. During inflammation, it is released into the blood. It exhibits a regulatory action on cell growth. Bertuccini et al. studied the effect of LF obtained from cattle on cells in vitro. They showed that the protein has antibacterial properties by reducing the secretion of proinflammatory cytokines (TNF-α, IL-8, and IL-6) and an insignificant bacterial adhesive property [108]. LF is present in neutrophils, the largest population of granulocytes, and its synthesis may increase during bacterial infections [109]. Sherman et al. additionally indicate that LF may reduce the severity of NEC (necrotizing enterocolitis) in infants through anti-inflammatory and supportive effects on the child's immune system [110]. Embleton et al. also describe the immunomodulatory effect of the compound [111]. MacManus et al. investigated the mechanism of LF action on inflammation (DSS—dextran sodium sulphate) in animal models. They showed that LF acts on T cells (Treg CD4+) by modulating inflammation, which has anti-inflammatory effects [112]. Biviano et al. studied the effect of LF on IBS (Irritable Bowel Syndrome) by administering *Bifidobacterium longum* in combination with LF to patients for 2 weeks, after which they observed a reduction in their symptoms (abdominal pain) [113].

#### 3.6.2. Colostrinin (CLN)

Colostrinin is a compound of proline-rich polypeptides, up to about 22%, found in the colostrum of milk. CLN has antiviral properties by activating leukocytes to produce interferon (INF). It also has an immunomodulatory action by activating the maturation of thymocytes and their differentiation and inhibits the production of autoantibodies [114]. Therefore, its application can be of crucial importance in autoimmune diseases. In addition, it can improve cognitive function, which can be helpful during Alzheimer's treatment [115]. It also has the ability to modulate cytokine secretion and is anti-inflammatory [116]. It may also reduce the level of reactive oxygen species (ROS) [117, 118].

#### 3.6.3. Glycomacropeptide (GMP)

This is a peptide with hydrophilic properties, derived from κ-casein. It has sialic acid in its composition. GMP has anti-inflammatory properties by inhibiting the expression of IL-17 and IL-2. It has an antibacterial effect reducing bacterial epithelial adhesion. Requena et al. analysed intestinal GMP and its immunomodulatory possibilities. The study was conducted on animal models. They observed that GMP has anti-inflammatory effects on the intestines through a modulating effect on lymphocytes. It also has properties that limit the secretion of INF-*γ* and TNF-*α* [119]. Lopez-Posadas et al. also studied the effect of GMP on enteritis. GMP activity was observed in a rat colitis model. They came to similar conclusions as those of Requena et al.; namely, the anti-inflammatory effect of GMP was associated with the effect of the peptide on Th1 and Th17 lymphocytes [120].

#### 3.6.4. Conjugated Linoleic Acid (CLA)

CLA is found primarily in products such as milk, dairy products, and ruminant meat and their derivatives. It can also be produced in the gut by bacteria in the *Bifidobacterium* family with linoleic acid (LA) and *α*-linolenic acid (LNA) [121]. It is a compound widely described in the literature, primarily due to its health-promoting properties. Shokryzadan et al. describe in their work that CLA may have an effect on lowering blood pressure in men with comorbid obesity. They suggest that CLA may exhibit blood-glucose-regulating properties and have beneficial effects on body weight control parameters, but they also indicate that there are many scientific reports that conflict with previously discovered properties of the compound [122]. Other researchers have come to similar conclusions describing the beneficial effects of CLA in animal model studies, but the same beneficial properties are not always observed in human studies [123, 124]. However, there are many scientific reports about its beneficial anti-inflammatory effect on the intestines. Bassaganya-Riera et al. show that CLA can modulate PPAR-*γ* in an in vivo study and thus acts as an anti-inflammatory [125]. In addition, they examined the immune response of patients with CD after administration of CLA. They showed that, in this group, CLA administration reduced the ability of T CD4+ and CD8+ to produce TNF-*α*, INF-*γ*, and IL-17, thus reducing disease activity [126].

#### 3.6.5. Phosvitin

Phosvitin occurs in eggs; more than half of the amino acids contained in its composition contain serine residues. It can reduce the formation of ROS and protect cells' DNA. It also has the ability to inhibit lipid peroxidation in cell membranes in the presence of excessive amounts of iron ions [127, 128]. Phosvitin also has anti-inflammatory properties. Xu et al. studied in vitro its effect on Caco-2 and HT-29 cells. The researchers induced inflammation through TNF-*α* and LPS. They observed that IL-8 secretion decreased after exposure to phosvitin, but the effect was dose-dependent. In addition, the compound inhibited the expression of other proinflammatory cytokines (IL-8 and IL-12) [129]. Lee et al. presented in their work the action of phosvitin increasing the phagocytic activity of macrophages in vitro [130]. Li et al. presented the effect of phosvitin on intestinal microflora in animal models. Increased amounts of *Bifidobacterium* and a decrease in *Anaerostipes* and *Prevotella* were observed in juveniles, with decreased amounts of *Mucispirillum* and *Helicobacter* in adults [131]. Fernández-Tomé et al. described in their work the effect of bioactive peptides on IBD. They point out the fact that despite the existence of many scientific reports describing their beneficial effects, their effects on the human body should be investigated, since most of them were studies using animal models [132].

#### 3.6.6. Taurine

This is a compound that belongs to the group of amino acids; it is formed from cysteine. It occurs primarily in products of animal origin. Significant amounts are found in fish and turkey meat. Sukhotnik et al. studied the effect of taurine on intestinal regeneration in animal models. They reported that the compound may reduce cell apoptosis, stabilize cell membranes, and exert beneficial effects on the regeneration of intestinal mucosa [133]. Taurine has antioxidant properties which may predispose to relief of inflammation [134].

## 4. Conclusions

Inflammatory bowel diseases are diseases with global reach. There is an increase in cases from year to year. They are predisposed by a number of different factors, including lack of physical activity and a Western type of diet (rich in saturated fatty acids and trans-fatty acids, or low in fibre). It has been shown that some food components may have protective effects in the development of these diseases and inhibit their active form. The most frequently mentioned components of such a diet are dietary fibre, especially the soluble fraction, essential fatty acids, probiotics, prebiotics, vitamins (D, E, and C), and zinc. Some of them have immunomodulatory functions, which support the human immune system in the fight against pathogens. A properly balanced diet should provide all the necessary components, for which the demand is increased during the active phase of the disease. However, further research is needed into the effect of some food ingredients on the course of the disease [16].

## Figures and Tables

**Table 1:** Composition, functions, and sources of dietary fibre according to the division into fractions [9].

Dietary fibre fraction	Composition	Main functions	Dietary sources
Water-soluble	(i) *β*-Glucans (ii) Pectin (iii) Mucilage (iv) Gums (v) Certain hemicelluloses	(i) Slows the passage time of the chyme (ii) Slows down the absorption of glucose into the blood (iii) Increases the excretion of fats and cholesterol in the faeces (iv) Nutrient for bacteria	Certain fruits and vegetables, dried legumes

Insoluble in water	(i) Cellulose (ii) Certain hemicelluloses (iii) Lignins	(i) Binds water, which increases the volume of chyme in the intestine (ii) Mechanically irritates the wall of the colon (iii) Binds excess hydrochloric acid in the stomach	Coarse milled cereal products

**Table 2:** Distribution of fatty acids and their dietary sources [17].

Type	Example	Dietary sources
Saturated fatty acids	Myristic acid Palmitic acid Lauric acid Arachidonic acid	Coconut oil, palm oil, and butter

Monounsaturated fatty acids	Oleic acid Crotonic acid Cetoleic acid	Olive oil, zero-erucic rapeseed oil

Essential unsaturated fatty acids	Omega-3 *α*-Linolenic acid Eicosapentaenoic acid Docosahexaenoic acid	Rapeseed oil, linseed oil, and fatty fish (e.g., salmon, mackerel, and sardines)
	Omega-6 Linoleic acid *γ*-Linoleic acid	Sunflower oil, grapeseed oil, and corn oil

**Table 3:** Vitamin D content of selected food products [39].

Food product	Vitamin D content *μ*g/100 g of the product
Hard cheese	0.2–0.6
Fermented milk drinks	0.01–0.03
Eggs	1.70
Butter	0.7
Fresh white halibut	5.00
Fresh salmon	13.00
Fresh mackerel	5.00
Fresh rainbow trout	13.60
Fresh herring	19.00

**Table 4:** *β*-Carotene content of selected vegetables [39].

Food product	*β*-Carotene content *μ*g/100 g of the product
Chard	4020
Broccoli	920
Beetroot	12
Endive	1586
Pumpkin	2974
Kale	5350
Fennel	2100
Carrot	9938
Cucumber	170
Parsley	5410
Parsley root	30
Tomato	640
Lettuce	1153
Potatoes	5

**Table 5:** *β*-Carotene content of selected fruits [39].

Food product	*β*-Carotene content *μ*g/100 g of the product
Banana	48
Peach	595
Apple	24
Raspberries	16
Melon	1100
Apricots	1523
Red currants	24
Strawberries	14

**Table 6:** Magnesium content of selected food products [39].

Food product	Magnesium content mg/100 g of the product
Milk	12
Pork shoulder	19
Chicken breast	33
Salmon	29
Wheat flour	10
Buckwheat	218
White rice	13
Brown rice	110
Wholemeal rye bread	64
White beans, dry seeds	169
Parsley	69
Red lentils seeds	71
Avocado	39
Melon	23
Almonds	269
Italian nuts	99
Pumpkin seeds	540
Sunflower seeds	359
